# Description of Some Specimens of Pleuro-Pneumonia Contagiosa*Presented at the semi-annual meeting United States Veterinary-Medical Association at Baltimore, March 20th, 1888.

**Published:** 1888-04

**Authors:** A. W. Clement

**Affiliations:** Baltimore


### Art. XII.—DESCRIPTION OF SOME SPECIMENS OF PLEURO-PNEUMONIA CONTAGIOSA.*

BY A. W. CLEMENT, V. S.
*Baltimore.*

Pleuro-pneumonia contagiosa is generally defined to be a contagious or infectious disease manifesting itself by certain characteristic lung lesions, and generally accompanied by a sero-fibrinous pleuritis ; the bronchial lymphatic glands are enlarged and softer than normal. The first case to which I will call your attention is one of left-sided acute pleuro-pneumonia ; it involves nearly the whole of the left lung, which, as you will see, is greatly enlarged, weighing forty-five pounds, while the right lung, which is healthy, weighs but six pounds. Thus you will see that the diseased lung contains thirty-nine pounds of exudate. The pleura is covered with exudate over the affected lung, and there is also a very abundant exudate about the organs in the

*Presented at the semi-annual meeting United States Veterinary Medical Association at Baltimore, March 20th, 1888.

superior mediastinum, a complication to which I shall call your attention later on. On section this lung presents the lesions characteristic of the disease. This is a very typical case and one upon which nobody who had ever seen a picture of a lung in this disease could possibly make a mis take. The lung is solid, the parenchyma is hepatized and there are varying shades of color, from a reddish pink to a dark red dependent upon the amount of blood present. The parenchyma is at the same time very moist (œdematous), and the interlobular tissue is filled with a jelly-like exudate opaque white in appearance. This varied color of the parenchyma compared with the white opaque color of the interlobular exudate gives rise to what is term the marbling of the lung. Very soon fibrous connective tissue appears in place of the interlobular exudate and also around the bronchous in the centre of the lobule. This new growth of interlobular connective tissue takes place beginning at the border of the lobule, so that between two lobules we have two narrow bands of connective tissue with the exudate still remaining between them. The connective tissue may continue to grow so as to entirely fill up the interlobular space, or as is more commonly the case this takes place in some parts, while in others the two bands separate and we have beginning necrosis. This separation continues in a certain direction and finally incloses a varying number of lobules, sometimes only one. When the ring is completed a sequestrum is formed and what we call chronic pleuro-pneumonia is the result. The bronchi resist the necrotic process longest, so that it is common to see a necrosed mass connected by bronchi with the healthy lung tissue. Finally the bronchi ulcerate through and are seen hanging from the wall of the cyst as fibrous prolongations; these bronchi are in the great majority of cases occluded, but in some cases a fine probe may be pushed through them. Finally these projections slough off and their ends becoming covered by the connective tissue which grows from the lungs surrounding the necrosed mass. Sometimes ulceration may take place into a bronchous at the border of the diseased portion before complete occlusion has taken place, and so establishes direct communication between the contents of the cyst and the healthy lung. This is the only way in which there is bronchial communication with a pleuro-pneumonia cavity. A bronchous is often seen leading toward a cyst, but instead of penetrating the wall it passes to the side of it. The new formation of peribronchial connective tissue, together with the pressure of the walls of the cavity, serve to completely occlude the bronchi.

What causes the necrosis is hard to say. One would think that the plugging of the blood-vessels leading to the part would be sufficient, but we see the same necrotic processes on a very much smaller scale in the bronchial glands.

Perhaps the virus which causes the disease has the property of producing necrosis, and at the same time of plugging the blood-vessels. As soon as sequestration is complete, disintregation of the necrosed mass takes place, and the cavity becomes filled with serum or more commonly with a purent material in which more or less of the sequestrum remains.

What I have said refers to typical pleuro-pneumonia. Unfortunately many cases which we are called upon to diagnose are by no means typical. For example, a herd of cows came to Baltimore from outside of the quarantine limits, from a region where pleuro-pneumonia was not supposed to exist. Soon after arrival two of them presented symptoms of acute contagious pleuro-pneumonia. One was slaughtered, and at the post-mortem the following lesions were found: Double pleurisy. Both lungs enlarged and solid on pressure. On section of the lungs the following appearances were noticed : There was interlobular exudation precisely like that seen in acute pleuro-pneumonia contagiosa, but it affected the greater portion of both lungs, and there was no hepatization of the parenchyma except in a very small area of one lung, and there the consolidation was of the broncho-pneumonia type. When number two was slaughtered much the same appearances were noticed but in this case only one lung was effected, and there were necrotic cavities in the interlobular conective tissue. The parenchyma was not consolidated.

Next comes a very interesting series of cases in which were scattered foci of consolidation throughout the lung. On section these solid areas presented typical pleuro-pneumonia cyst in the areas large enough to be seen easily with the naked eye ; these areas varied in size from two or three lobules to a single lobule, and from a single lobule down to foci the size of the head of a pin, many of which could be seen in a single lobule. These small foci had very much the appearance of tubucles, but on a closer inspection a small necrotic centre was to be seen, even in the smallest, and on microscopic examination these centres were seen to be composed of a few air-cells which had undergone microsis precisely like that in the larger cysts. Now there were cases of typical pneumonia in the same herd and the evidence in considering them as pleuro-pneumonia is that in some cases a whole lung was involved, in others a greater portion of a lobe, in others, a small group of lobules together with single lobules and small foci, varying in size from a pea to a pin's head in the same lobule. That the same characteristics were presented in all of them even to the smallest.

In conclusion I wish to call your attention to a few specimens which I think are very interesting and instructive. In case No. 1 we have acute pleuro-pneumonia contagiosa of one lung, and in addition to this an exudation and new growth of tissue surrounding the base of the trachea and extending back through the superior mediastinum, involving the lymphatic glands, the œsophagus and the posterior aorta This exuadation looks precisely like that in the lung, and the lymphatic glands present many foci of necrosis. Case No. 2 presents the same changes around the trachea, but in this instance extends upward so that it could be seen half way up the neck It also extends downward by the large bronchi into the substance of the lungs, where there is chronic pleuro-pneumonia involving a considerable area in one lung. Case No. 3 shows exudation and new growth of tissue in the superior mediastinum extending to the posterior border of the lung and involving all the organs in this region. The character of these changes shows the process to have been of some standing, while the only invasion of the lung tissue is at the root of both lungs in an area about half the size of a hen's egg, where there is undoubted contagious pleuro-pneumonia, the inter-lobular exudation being directly continuous with that in the superior mediastinum. The changes in the mediastinum are of much longer standing than those in the lungs and certainly point to the fact that we may have what is termed pleuro-pneumonia contagiosa without any lung lesions .

